# The therapeutic effect of silymarin in the treatment of nonalcoholic fatty disease: A meta-analysis (PRISMA) of randomized control trials

**DOI:** 10.1097/MD.0000000000009061

**Published:** 2017-12-08

**Authors:** Sheng Zhong, Yuxiang Fan, Qi Yan, Xingyu Fan, Bo Wu, Yujuan Han, Ying Zhang, Yong Chen, Huimao Zhang, Junqi Niu

**Affiliations:** a Department of Neurosurgery, The First Hospital of Jilin University; b Clinical College, Jilin University; c Hepatopancreatobiliary Medicine Department, The First Hospital of Jilin University, Changchun; d Basic Medical College, Qiqihar Medical University, Qiqihar; e Department of Radiology, The First Hospital of Jilin University, Changchun, China.

**Keywords:** combination therapy, monotherapy, nonalcoholic fatty liver disease, pharmacological therapy, silymarin

## Abstract

**Background:**

Silymarin (SIL) is an active extraction of the silybum marianum, milk thistle, which is an ancient medicinal plant for treatment of various liver diseases for centuries. This study is to assess the therapeutic effect of SIL in the treatment of nonalcoholic fatty liver disease through meta-analysis.

**Methods:**

Published randomized controlled trials (RCTs) were included from electronic databases (PubMed, Embase, Cochrane library, Web of Science, and so forth). Cochrane handbook was applied to evaluate the methodological quality. All statistical analyses were directed by Revman 5.3 software, and statistical significance was defined as *P* < .05.

**Results:**

Eight RCTs involved 587 patients were included in this study. The results showed that SIL reduced the AST and ALT levels more significantly than the control group (AST UI/L: MD = −6.57; 95% CI, −10.03 to −3.12; *P* = .0002; ALT UI/L: MD = −9.16; 95% CI, −16.24 to −2.08; *P* = .01). Compared with other interventions, there were significant differences decreasing AST and ALT levels when SIL was used alone (AST UI/L: MD = −5.44; 95% CI, −8.80 to −2.08; *P* = .002; ALT UI/L: MD = −5.08; 95% CI, −7.85 to −2.32; *P* = .0003).

**Conclusion:**

SIL has positive efficacy to reduce transaminases levels in NAFLD patients. SIL can be an encouraging and considerable phytotherapy for NAFLD patients.

## Abstract 2

Supplemental Digital Content is available in the text

## 1 Introduction

Nonalcoholic fatty liver disease (NAFLD) is a prevalent metabolic disorder known for largely macrovascular steatosis in liver with little consumption of alcohol. Current statistics facilitate the estimation that 27% Asian population and 32% of population in Middle East are afflicted with the disease, especially in overweight people and type 2 diabetic patients, whose morbidity reaches 58% and 74% respectively.^[1,2]^ The initiation of NAFLD appears to closely relate with obesity and type II diabetes, while progressively predisposing to nonalcoholic fatty steatohepatitis (NASH) and liver cirrhosis probably due to insulin resistance (IR) and genetic susceptibility.^[3]^ NAFLD covers a complicated spectrum of hepatic disorders ranging from simple steatosis to NASH, cirrhosis with complication of liver dysfunction and heptaocellular carcinoma (HCC). Compelling evidences indicate that NAFLD has become the third leading cause of HCC. NAFLD patients still have potential risks to develop malignancy and HCC in the absence of cirrhosis.^[4]^ Radiologic surveillance and screening for HCC are warranted. Meanwhile, if incipient NAFLD progresses, the ensuing cardiovascular diseases (CVD) remain the main reason for the high mortality of NAFLD patients.^[5]^

However, multifactorial reasons for the etiology make diagnosing and treating NAFLD a challenge, especially the management of the diseases. Initially, asymptomatic patients with NAFLD might ignore their health issues and it would progress to NASH before manifestation.^[6]^ Only a few consistent approaches are available concerning the treatments, which depend on pharmacological interventions and lifestyle modifications mainly. It is recommended to exert physical exercise and have nutrition consults in order to achieve moderate weight-control and improve insulin sensitivity. Although multiple drugs such as thiazolidinediones, metformin, lipid-lowering agents, and antioxidants have been studied, an expected one can hardly be found because of severe side effects and limitations.^[7–9]^ Thus, it urgently requires developing a successful drug for NAFLD patients.

Some studies shed light on the phytotherapy against NAFLD, among which silymarin (SIL) drives people to investigate most. SIL, extracted from the fruit and seeds of the silybum marinum (milk thistle), contains a family of flavonolignans (silybin, isosilybin, silychristin, isosilychristin, and silydianin) and a flavonoid (taxifolin), among which silybin accounts for 50% to 70% of the extraction and is identified as major biologically active component. It has been reported as a potent therapeutic component for treatment of various liver diseases for centuries.^[10]^ Several studies in vitro and animal models have credited the SIL's therapeutic role treating NAFLD to its antiinflammatory, antioxidant, and antifibrotic properties. Recently, SIL extract tablets treated fatty liver disease in several clinical trials, whose results showing decreased hepatic enzymes levels in serum, especially ALT, indicated that SIL could partially restore the liver's function and mitigated NASH patients’ symptoms.^[11]^ Furthermore, there were few side effects when administrating with therapeutic dosage.^[12]^ Therefore, SIL could be a promising herbal regimen to treat NAFLD patients.

Plentiful clinical trials have been conducted to verify the efficacy of using SIL alone or in combination with other agents in treatment of NAFLD. However, these studies provided controversial conclusions of SIL's efficacy and safety. Here, this meta-analysis evaluated the therapeutic effect by analyzing eligible studies and statistics, relevant indices such as hepatic enzymes to provide guidelines for clinical decisions and further researches.

## 2 Materials and methods

This study was approved by the Ethics Committee of First Hospital of Jilin University. This meta-analysis was performed as listed steps: planed search strategy, selected study according to inclusion and exclusion criteria, assessed the quality of studies included, extracted the data, defined the outcomes, and analyzed the data.

### 2.1 Search strategy

**Figure 1:**
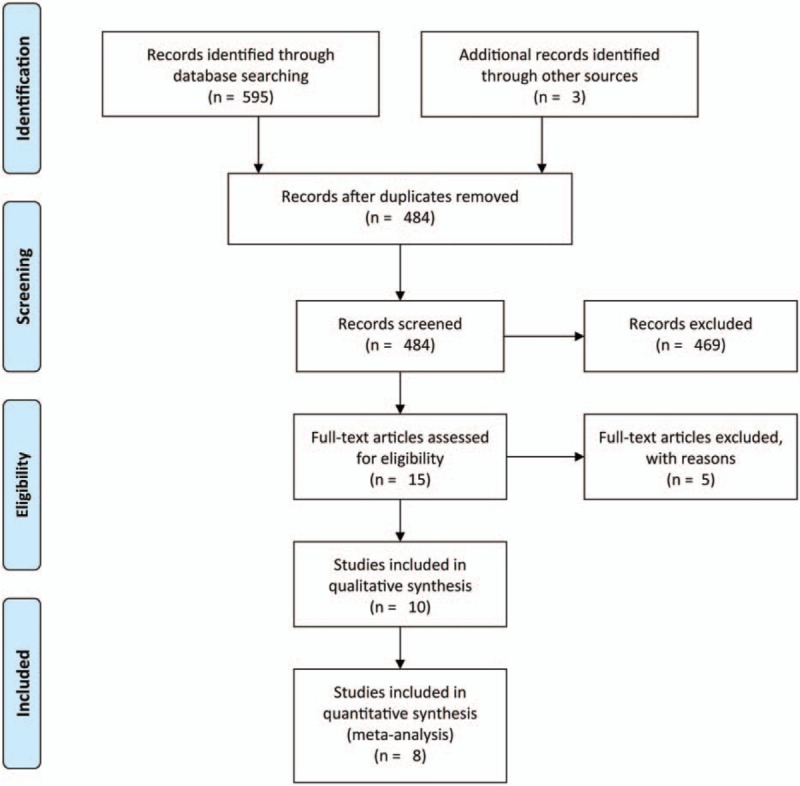
Flowchart of the study selection.

The process of article selection is shown in Fig. 1. PubMed, Embase, Cochrane, Web of Science databases for full article of randomized control trials between January 1996 and June 2017, as well as Chinese databases like SinoMed, CNKI (Chinese journal full-text database), VIP database, and Wan Fang database were searched. The searching procedure was done and cross-checked by 2 reviewers (SZ and YF) independently. Search terms were SIL, silymarin, karsil, legalon, carsil, NAFLD, nonalcoholic fatty liver disease, NASH, nonalcoholic steatohepatitis, fatty liver, fatty liver disease, randomized controlled trial (RCT), and controlled clinical trial. The search strategy is affiliated in Appendix 1.

### 2.2 Study selection

The eligibility assessment was applied by screening the titles and abstracts before checking full text of the articles. The selection of the all studies was independently made by 2 reviewers based on the inclusion criteria. Disputes on whether an article should be included were resolved by a third reviewer.

### 2.3 Inclusion and exclusion criteria

The included articles should meet the following criteria:

The patients: *Inclusion criteria*: Patients inflicted with NAFLD confirmed by performing liver sonography, radiography, and elevated liver enzymes AST and ALT level; patients daily ethanol intake less than 20 g/day; patients’ ages older than 18. *Exclusion criteria*: Patients having any history of alcohol abuse, diabetes, severe cardiac, pulmonary, renal or psychological problems, positive pregnancy test, positive results for tests of HBV and HCV, autoimmune hepatitis, Wilson disease, hemochromatosis, and alpha-1 antitrypsin deficiency; patients suffering from alternative substances abuse, zero using of drugs such as statin, fibrate, NSAID, acetaminophen, warfarin, metronidazol, antipsychotics, and antihistamines.

The design of studies and comparisons: *Inclusion criteria*: Randomized controlled clinical trials published as formal papers; SIL monotherapy group and SIL combination group were labeled as experimental group; placebo and the controlled agents should be in the same shape, odor, and schedule from the same pharmaceutical company. *Exclusion criteria*: Low quality clinical control studies, case reports, reviews, letters, and the trials with patients less than 10 and the treating time less than 1 week criteria were excluded; studies with standard deviations and confidential intervals of the tested parameters not reported and an absence of key information such as the sample size, HR, 95% CI, and *P*-value were excluded.

The outcomes: Biochemical markers alanine transaminase (ALT), aspartate transaminase (AST), total cholesterol (TC), and triglyceride (TG) were measured automatically to judge the liver's injury and lipid profile accumulating in blood. The indices were presented by the concentration in patients’ blood.

### 2.4 Quality assessment

The Cochrane handbook was applied to assess the trial quality. The following details were extracted: blinding; method of randomization including the stratification factor of the number of randomly assigned patients, exclusion from the analysis by arm, patient follow-up time if possible by arm, and number of patients lost to follow-up by arm. The assessment outcomes are shown in Appendix 2.

### 2.5 Data extraction

All of the data were extracted independently by 2 reviewers according to the selection criteria (SZ and YF), any disagreements were discussed and documented. When the extracted data were not uniform, consults were needed to settle the disagreements and to make a final determination. All trials included in this study contained the following data: first author's name, published year, type of study, country of origin study, percentage of men, the number of patients, average ages, interventions, and outcomes.

### 2.6 Outcome definition

The data from each included study were recorded by 2 investigators independently. Studies taking SIL alone as experimental agent were considered as SIL monotherapy, while those using SIL jointed with other drugs were labeled as SIL combination. Biochemical markers ALT, AST, TC, and TG were measured to evaluate the liver's function and lipid deposition in blood. These indices were presented by the concentration in patients’ blood.

### 2.7 Statistical analysis

Review Manager (RevMan 5.3) was used to assess pooled mean deviation (MD) and standard deviation (SD) for continuous outcomes. 95% Confidence interval (95% CI) was regarded as effective size in the combined analysis. Chi-square and *I*^2^ tests were performed to assess the heterogeneity. The fixed-effect model was applied when *P* > .1 or *I*^2^ < 50% considered as homogeneous, and the random-effect model was more eligible when *I*^2^ > 50%. Statistical significance was defined as *P* < .05.

## 3 Results

### 3.1 General characteristics of included studies

The characteristics of studies included are clarified in Table 1. After eligibility assessment, we finally obtained 8 records: 6 in English and 2 in Chinese.^[11,13–19]^ Among these records, the study of Hajiaghamo et al^[17]^ included 2 control groups: metformin group and pioglitazone group, thus, we divided this study into 2 parts: Hajiaghamo et al 2012 met. and Hajiaghamo et al 2012 pio. when analyzing. The publication year ranged from 2005 to 2015, and the case load varied from 55 to 100. A total of 587 patients were included in the analysis. SIL monotherapy group and SIL combination therapy group were treated as experimental group. By way of contrast, placebo and antimetabolic disorder regimens, namely pioglitazone and metformin, simvastatin, vitamin E, and Gankangyin, which is a traditional Chinese regimen, were set being controlled drugs. We analyzed the baseline data of the patient of included studies, showing there were no differences between 2 groups, and all the usage of medicine met the inclusion criteria with good compatibility. The characteristics of the 8 included studies are listed in Table 1.

**Table 1:**
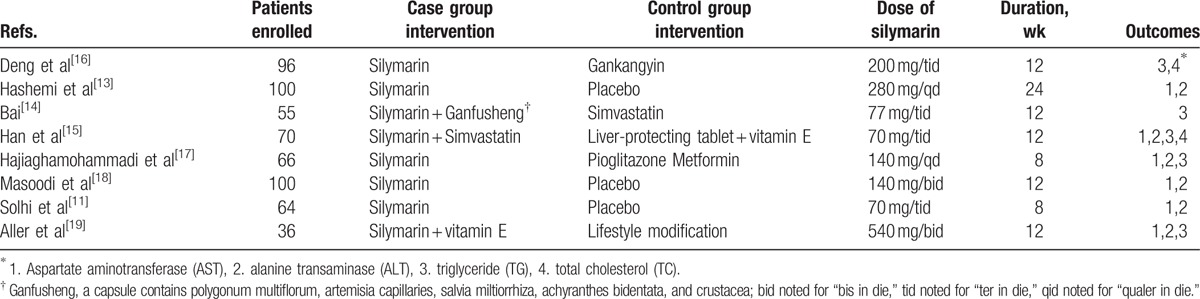
Characteristics of the studies included in the meta-analysis.

To summarize, all participants enrolled in the RCTs contained Asian (Iranian, Chinese) and European (Spanish, Italian). The median age was approximately 40. Bai^[14]^ and Solhi et al^[11]^ intervened the participants’ lifestyle and recommended low-calorie diets and physical exercise to control weight. It was noticed that 4 trials had viewed NASH as a representative from NAFLD and recruited NASH patients for their studies.

### 3.2 Meta-analysis

#### 3.2.1 The effect of silymarin on liver function in NAFLD patients

**Figure 2:**
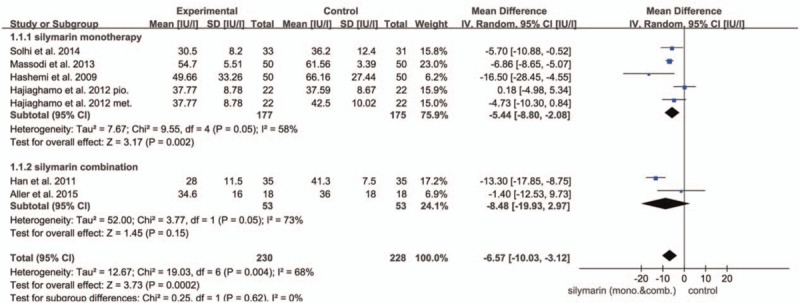
Forest plot of the AST in the meta-analysis and subgroup analysis. There was a significant difference between the 2 arms (AST UI/L: MD = −6.57; 95% CI, −10.03 to −3.12; *P* = .002).

AST was reported by 6 trials. The *I*^2^ value being 68% indicated indispensable heterogeneity. The selected random-effects model showed that the reduction of AST in SIL treating group (the SIL combination group and SIL comparing to other interventions group) was more significant than that of corresponding drugs in control group (AST UI/L: MD = −6.57; 95% CI, −10.03 to −3.12; *P* = .0002) (Fig. 2). It was conceivable to conclude that SIL therapy maintained superior efficacy in lowering AST level.

**Figure 3:**
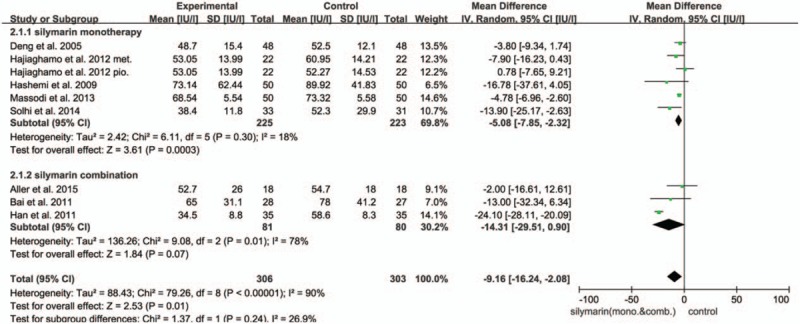
Forest plot of the ALT in the meta-analysis and subgroup analysis. There was a significant difference between the 2 arms (ALT UI/L: MD = −9.16; 95% CI, −16.24 to −2.08; *P* = .01).

ALT was taken as an essential index by 8 trials. Notably, the heterogeneity was high (*I*^2^ = 90%) that a random-effect model was employed. It revealed that there was significant difference in decreasing ALT level between the SIL treating group and control group, which suggested SIL could also play a pivotal role in hepatic protection by reducing ALT level in serum (ALT UI/L: MD = −9.16; 95% CI, −16.24 to −2.08; *P* = .01) (Fig. 3).

#### 3.2.2 The effect of silymarin on blood lipids in NAFLD patients

**Figure 4:**
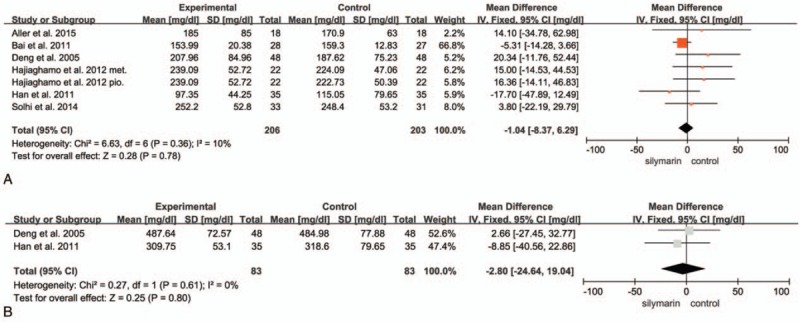
(A) Forest plot of TG. There was no significant difference between the 2 arms (*P* = .78). (B) Forest plot of TC. There was no significant difference between the 2 arms (*P* = .80).

TG and TC were measured by 7 trials and 2 trials, respectively. TG trials involved 409 patients, with 206 in experimental group and 203 in control group. The heterogeneity was not evident (*I*^2^ = 10%; *P* = .36), but there was no statistical significances between the SIL group and the control group (*P* = .78) (Fig. 4A). A total of 166 patients were involved in the assessment of TC with 83 patients in each group. Similarly, there was no heterogeneity (*I*^2^ = 0; *P* = .61) and the statistical significance did not exist either (*P* = .80) (Fig. 4B). In conclusion, neither TG nor TC dropped obviously after SIL treatments in comparison with their controls.

### 3.3 Subgroup analysis

In addition, we performed subgroup analysis grouped by whether SIL was used alone. Intriguingly, there was a significant difference in terms of AST level (*P* = .002). It turned out that the heterogeneity diminished (*I*^2^ = 58%) in the SIL monotherapy group, and this group appeared to reduce AST level more effectively than other drugs or lifestyle interventions (AST UI/L: MD = −5.44; 95% CI, −8.80 to −2.08; *P* = .002) (as shown in Fig. 2). The SIL monotherapy group achieved the reduction in the ALT level significantly (ALT UI/L: MD = −5.08; 95% CI, −7.85 to −2.32; *P* = .0003). We also found that in both AST and ALT analysis, the SIL monotherapy group could have attained more robust therapeutic efficacy than SIL combination group (AST UI/L: MD = −8.48; 95% CI, −19.93 to 2.97, *P* = .15; ALT UI/L: MD = −14.31; 95% CI, −29.51 to 0.90; *P* = .07), so that we assume that the SIL monotherapy group exhibit improved therapeutic efficacy than combination group (as shown in Fig. 3). Nevertheless, when excluding Aller et al,^[19]^ SIL combination group show a favorable result than SIL monotherapy group. Neither of the 2 groups resulted in significantly lower TG or TC level.

### 3.4 Sensitivity analysis

We excluded each study individually to verify the reliability of our conclusions. None of the significance altered in AST, TG, and TC. To further elucidate the substantially high heterogeneity detected in meta-analysis for ALT, we decided to conduct a sensitivity analysis. The result suggested that the study carried out by Han et al^[15]^ contributed greatly to the evident heterogeneity. After the study was excluded, the statistical heterogeneity *I*^2^ value dramatically reduced to 0% (shown in Appendix 3). It was reasonable to approve the SIL therapeutic effects regarding the improvement of ALT level (*P* < .0001).

## 4 Discussion

Given that overnutrition and inactivity has become increasingly regular, nonalcoholic fatty liver disease (NAFLD) is one of the most common and emerging chronic liver disorders worldwide.^[20–23]^ Obesity plagues the world and nations, which also aggravates the problems. Liver serves as a principle organ catabolizing the lipids, glucose, proteins, and other nutrients in the body, it gives rise to systematic metabolic disorders when excessive intake overwhelms the liver. Therefore, it is necessary to develop promising and potent drug in treatment of NAFLD.^[24,25]^

This meta-analysis consists of 8 randomized clinical trials including 587 patients with the intention to illustrate SIL clinical value. It was observed that SIL could relatively revive the liver by lowering not only the AST level but also ALT level comparing to controlled regimens, which was in accordance with previous researches.^[12,26]^ Additionally, regarding to AST and ALT level, the SIL monotherapy group could have attained more robust therapeutic efficacy than SIL combination group (AST UI/L: MD = -8.48; 95% CI, 19.93 to −2.97, *P* = .15; ALT UI/L: MD = −14.31; 95% CI, −29.51 to 0.90; *P* = .07). Previous study has already proved that improvement in transaminases demonstrated reviving of liver function in NAFLD patients, it also predicted a better prognosis and a lower incidence of progression to either liver cirrhosis or HCC.^[25,26]^ In summary, SIL is a promising drug to improve liver function of NAFLD patients. However, there are less significant results in restoring TG and TC (TG, *P* = .78; TC, *P* = .80). As we all know, to improve the liver function is a long-term process and the SIL duration and dosage are also crucial for final outcome. It is fair to believe that TG and TC will eventually improve treated with the rational dosage regimen in the future researches.

SIL, as an insulin sensitizer, could generate a moderate increase of the expression of glucose transporter type 4 (GLUT4), thereby ameliorating the IR via insulin receptor substrate 1 (IRS-1)-PI3K-Akt pathway.^[27]^ It was reported that SIL had shown encouraging effects in reducing TNF-ɑ, IL-1β, and IL-6 in animal models.^[28,29]^ Patients with NAFLD benefited from SIL scavenging oversupplies of fatty acids and oxidative stress. In the light of defense against free radicals, SIL rebalanced the high fatty acids catabolism in response to lipids accumulation and oxidative stress induced by lipids peroxidation via peroxisome proliferator-activated receptors (PPARs) and nuclear factor kappa B (NF-κB) pathways.^[30,31]^ Additionally, SIL possesses the capacities to stabilize the hepatocyte membrane structure and manipulate the membrane permeability in the presence of the toxicity, as well as to retard the transformation from hepatic stellate cell to myofibroblasts resulting in liver fibrosis and cirrhosis.^[32,33]^

The elevation of AST and ALT level represents the prevailing abnormalities in NAFLD patients.^[2]^ There was more extensive reduction in ALT level than that in AST after SIL treatments, because the original level of ALT was far higher than the AST leaving the more for ALT to decline accordingly. It had become the norm that NAFLD patients AST/ALT ratio <1. But this ratio increased when cirrhosis presented. Under the premise that little correlation between the transaminase levels and the histological diagnosis had been noticed previously, these liver enzymes levels could be a feasible and noninvasive clue to general liver injury but more elaborate histological and radiological tests should be ordered when requiring more specificity and sensitivity.^[34–36]^ The results in subgroup analysis showed the superiority of SIL monotherapy group. However, when excluding Aller et al,^[19]^ SIL combination group show a favorable result than SIL monotherapy group. Therefore, SIL used as monotherapy or combined with other drugs except vitamin E (the therapy regimen in Aller et al^[19]^) was recommended for the future research.

Damaged hepatocytes hinted by aberrant aminotransferases level required agents to recover proceeding fatty acids oxidation. SIL might enable the patients to reprocess the deposited or excessive lipid profile, which was reported by Deng et al,^[16]^ Han et al,^[15]^ and Bai,^[14]^ even though the outcome effects had rather low value of significant differences in TG and TC level. In this regard, it needed more RCTs and further basic researches to prove. Furthermore, Han et al^[15]^ also reaped the benefits treating NASH patients with SIL and simvastatin. SIL combined with statins therapy showed the improved efficacy with mild counteraction against the statins’ side effects elevating aminotransferases level and could improve the patients’ condition.

Hypocaloric diet and physical exercises implementation into lifestyle, conducted by Solhi et al^[11]^ and Bai,^[14]^ would enhance the lipid catabolism and pharmacological therapy theoretically,^[37]^ but poor estimate of daily calorie consumption from each patient had been reported. Therefore, the lifestyle modifications and dietary alterations as adjuvant therapy required reconsideration to evaluate their contribution.

It was interesting that patients from Hashemi et al,^[13]^ Han et al,^[15]^ Bai,^[14]^ and Solhi et al^[11]^ were diagnosed as NASH, an advanced stage of NAFLD. It would be available to assess the part SIL played in halting the NASH progression. But some lesions might be irreversible in NASH patients, of which the drug efficacy could marginally be improved, whereas it did not receive the attention that deserved.^[27–30,32]^ Currently, employments of insulin sensitizers or antioxidant vitamin E for certain duration were highly debated.^[37,38]^ A bundle of studies reported the beneficial outcomes in silymarin-treated NAFLD patients. The decrease in aminotransferases levels, the drop with respect to the lipid profile and the IR improvements indicated its effectiveness and promising applications in NAFLD. Velussi et al^[25]^ reckoned that SIL (silymarin 600 mg/day, 12 months) enhanced the endogenous and exogenous insulin sensitivities directly, which suggested an interesting regimen for NAFLD. Hajaghamohammadi et al^[26]^ performed a well-designed randomized clinical trial (silymarin 140 mg/day, 2 months) and observed the profound reduction in mean serum ALT level from 103.1 to 41.4 IU/mL, while AST level from 53.07 to 29.1 IU/mL. Loguercio et al^[12]^ confirmed the positive manipulation of liver enzymes with SIL complex (silybin 94 mg + vitamin E 90 mg + phospholipids 194 mg for each pill, at a dose of 2 pills/day for 12 months).

SIL is a safe and well-tolerance drug,^[19,39]^ and the results in this meta-analysis showed that SIL was not dose-dependent. SIL could be a preferential option for NAFLD patients, especially NASH, in contrast with antimetabolic disorders agents and hepatoprotective drugs. It might be an optimal therapy if conjugated with drugs against metabolic syndrome, for example, statins, and lifestyle modifications as supplements. Based on our clinical experience and the results in this meta-analysis, the regimen in Han et al^[15]^ (silymarin 70 mg/tid 12 weeks + simvastatin) and Masoodi et al^[18]^ (silymarin 140 mg/bid 12 weeks) were strongly recommended because of their robust therapeutic effect and less side effect events.

To the best of our knowledge, this study has reported the most updated information in this field. Compared with previous studies, all the studies included in this meta-analysis are high quality studies, all the low-quality trials and unqualified patients (HCV infector and diabetic patients) were removed, thus this meta-analysis attained the evidence level 1. And the conclusions provided in this study are the most convincing and solid.

There were several unavoidable limitations in this study. Primarily, fewer studies included might introduce some biases. Therefore, it is necessary to perform prospective, standardized, multicenter, and larger sample sized RCTs with unbiased methods in the future. The conclusions in histological changes had scarcely reached, because it might be committed sampling errors and introduce some subjective bias when scoring pathologically. NAFLD covered a wide range of chronic liver diseases but the majority of the studies take NASH as a main objective to research rather than the each stage of NAFLD. Lastly, as subgroup analysis and sensitive analysis were done, variations in the backgrounds of the studies might impact the ultimate effects.

## Supplementary Material

Supplemental Digital Content
