# Supplementary material for: The therapeutic effect of silymarin in the treatment of nonalcoholic fatty disease: A meta-analysis (PRISMA) of randomized control trials
Source: Medicine (Baltimore). 2017 Dec 8;96(49):e9061. doi: 10.1097/MD.0000000000009061 (PMC5728929; doi:10.1097/MD.0000000000009061)

**Appendix 1**

**Search strategy**

Sum=595

Cochrane：20

#1 Nonalcoholic Fatty Liver Disease:ti,ab,kw (Word variations have been searched)

#2 MeSH descriptor: [Non-alcoholic Fatty Liver Disease] explode all trees and with qualifier(s): [Drug therapy - DT, Therapy - TH]

#3 Nonalcoholic Steatohepatitis

#4 Fatty Liver, Nonalcoholic

#5 Liver, Nonalcoholic Fatty

#6 Nonalcoholic Fatty Liver

#7 Nonalcoholic Steatohepatitis

#8 Steatohepatitis, Nonalcoholic

#9 silymarin or silimarin

#10 karsil

#11 legalon

#12 carsil

#13 #1 or #2 or #3 or #4 or #5 or #6 or #7 or #8

#14 #9 or #10 or #11 or #12

#15 #13 and #14

Pubmed:57

#1 Search ((((((Nonalcoholic Fatty Liver Disease) OR Nonalcoholic Steatohepatitis) OR Fatty Liver, Nonalcoholic) OR Liver, Nonalcoholic Fatty) OR Nonalcoholic Fatty Liver) OR Nonalcoholic Steatohepatitis) OR Steatohepatitis, Nonalcoholic

#2 Search (((silymarin) OR karsil) OR legalon) OR carsil

#3 #2 AND #1

Web of Science:35

#1 TS=Nonalcoholic Fatty Liver Disease OR TS=Nonalcoholic Steatohepatitis OR TS=Fatty Liver, Nonalcoholic OR TS=Liver, Nonalcoholic Fatty OR TS=Nonalcoholic Fatty Liver OR TS=Nonalcoholic Steatohepatitides OR TS=Steatohepatitides, Nonalcoholic OR TS=NAFLD

#2 TS=silymarin OR TS=karsil OR TS=legalon OR TS=carsil

#3 #1 AND #2

Embase:152

#1 'nonalcoholic fatty liver disease'/exp

#2 steatohepatitis, AND nonalcoholic

#3 liver, AND nonalcoholic AND fatty

#4 NAFLD

#5 silymarin OR silimarin OR karsil OR legalon OR carsil

#6 #1 OR #2 OR #3 OR #4

#7 #5 AND #6

Chinese Database:

Wanfang:86

#1 ‘Nonalcoholic Fatty Liver Disease’ OR ’ Nonalcoholic Steatohepatitis’ OR ’ Nonalcoholic Fatty Liver’ OR ‘NAFLD’

#2 silymarin

#3 #1 AND #2

CNKI:82

#1 ‘Nonalcoholic Fatty Liver Disease’ OR ’ Nonalcoholic Steatohepatitis’ OR ’ Nonalcoholic Fatty Liver’ OR ‘NAFLD’

#2 silymarin

#3 #1 AND #2

Sinomed:133

#1 ‘Nonalcoholic Fatty Liver Disease’ OR ’ Nonalcoholic Steatohepatitis’ OR ’ Nonalcoholic Fatty Liver’ OR ‘NAFLD’

#2 silymarin

#3 #1 AND #2

**Appendix 2**

**Study quality ass****essment graph**


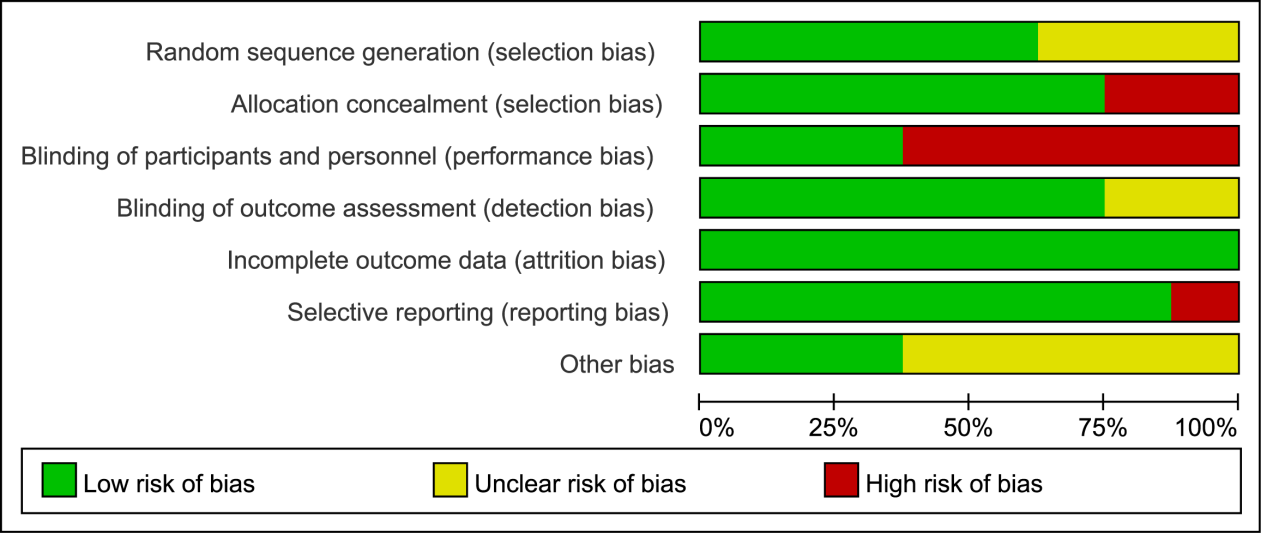


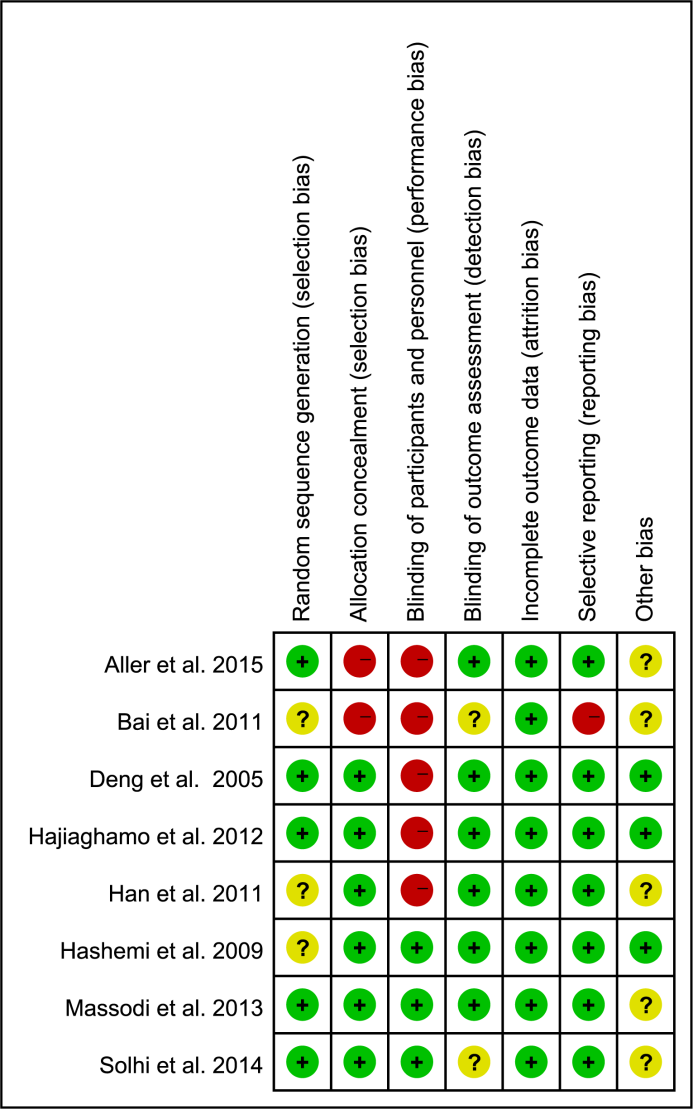


**Appendix 3**

**Forest plot of ALT excluding HAN 2011**


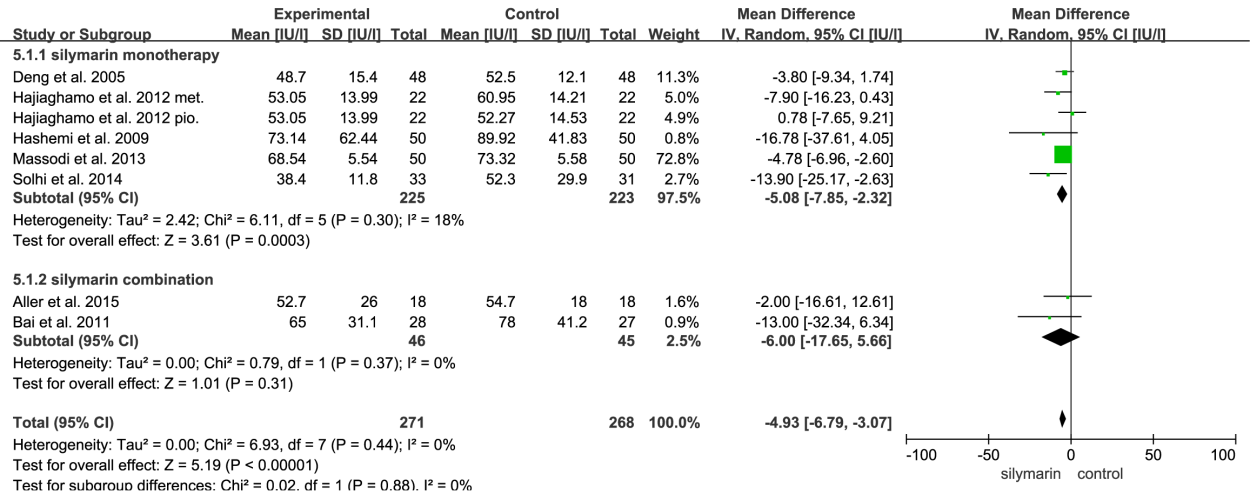

Supplement: Supplemental Digital Content [file medi-96-e9061-s001.doc]
